# Semen Microbiome Biogeography: An Analysis Based on a Chinese Population Study

**DOI:** 10.3389/fmicb.2018.03333

**Published:** 2019-01-31

**Authors:** Zhanshan (Sam) Ma, Lianwei Li

**Affiliations:** ^1^Computational Biology and Medical Ecology Lab, State Key Laboratory of Genetic Resources and Evolution, Kunming Institute of Zoology, Chinese Academy of Sciences, Kunming, China; ^2^Center for Excellence in Animal Evolution and Genetics, Chinese Academy of Sciences, Kunming, China

**Keywords:** semen microbiome, biogeography, inter-subject heterogeneity, DAR (diversity-area relationship), beta-diversity, RIP (ratio of individual to population accrual diversity), LRD/LGD (ratio of local to regional/global diversity)

## Abstract

Investigating inter-subject heterogeneity (or spatial distribution) of human semen microbiome diversity is of important significance. Theoretically, the spatial distribution of biodiversity constitutes the core of *microbiome biogeography*. Practically, the inter-subject heterogeneity is crucial for understanding the normal (healthy) flora of semen microbiotas as well as their possible changes associated with abnormal fertility. In this article, we analyze the scaling (changes) of semen microbiome diversity across individuals with DAR (diversity-area relationship) analysis, a recent extension to classic SAR (species-area relationship) law in biogeography and ecology. Specifically, the unit of “area” is individual subject, and the microbial diversity in seminal fluid of an individual (area) is assessed *via* metagenomic DNA sequencing technique and measured in the Hill numbers. The DAR models were then fitted to the accrued diversity across different number of individuals (area size). We further tested the difference in DAR parameters among the healthy, subnormal, and abnormal microbiome samples in terms of their fertility status based on a cross-sectional study of a Chinese cohort. Given that no statistically significant differences in the DAR parameters were detected among the three groups, we built unified DAR models for semen microbiome by combining the healthy, subnormal, and abnormal groups. The model parameters were used to (i) estimate the microbiome diversity scaling in a population (cohort), and construct the so-termed DAR profile; (ii) predict/construct the maximal accrual diversity (MAD) profile in a population; (iii) estimate the pair-wise diversity overlap (PDO) between two individuals and construct the PDO profile; (iv) assess the ratio of individual diversity to population (RIP) accrual diversity. The last item (RIP) is a new concept we propose in this study, which is essentially a ratio of local diversity to regional or global diversity (LRD/LGD), applicable to general biodiversity investigation beyond human microbiome.

## Introduction

Similar to other human microbiome habitats such as gut, vaginal, or breast milk, human seminal fluid also hosts a microbiome including several hundreds of bacterial species per individual with various levels of abundances (Kiessling et al., 2008; Moretti et al., 2009; De Francesco et al., 2011; Hou et al., 2013; Weng et al., 2014). The seminal microbiome, just like other human microbiomes, is highly personalized. If we considered a cohort or population of men, their semen microbiomes are independent in ecological time in general, and each individual is not unlike an island available for microbes to invade and/or inhabit. Similar scenarios have been investigated extensively in macro-ecology of plants and animals since the 1960s, started with MacArthur and Wilson's (1967) island biogeography. The biogeography studies the spatial and/or temporal distribution of biodiversity and is a foundation of the modern conservation biology and biodiversity conservation in large. It has been widely recognized that seminal microbiome is implicated, at least in some of the male infertilities (Kiessling et al., 2008; Moretti et al., 2009; De Francesco et al., 2011; Domes et al., 2012; Hou et al., 2013; Weng et al., 2014). Therefore, investigating the biogeography or spatial distribution of seminal microbiome diversity is necessary for deep understanding the seminal microbiome as well as their implications for male infertility.

Prior to recent large-scale DNA sequencing studies of seminal microbiome samples (e.g., Hou et al., 2013; Weng et al., 2014), most studies on seminal microbes were focused on acute and chronic microbial infections, either based on PCR, microscopic or artificially culture-based methods (Keck et al., 1998; Henkel et al., 2006; Kiessling et al., 2008; Lbadin and Ibeh, 2008; Ochsendorf, 2008; Moretti et al., 2009; Akutsu et al., 2012; Domes et al., 2012), and majority of the early studies were conducted to explore the relationship between infections and male infertility. It was reported that infectious etiologies cause about 15% of male infertility cases (Diemer et al., 2003; Weng et al., 2014). The adoption of NGS (next generation sequencing) technologies have lead to significant advances in understanding the semen microbiome, because it greatly expanded our capability to detect virtually all bacteria in seminal fluid with rather low cost. Since the cataloging the semen microbes is not limited to infectious or opportunistically infectious microbes anymore, the NGS-based metagenomic technology and associated bioinformatics analyses have made the examination of the whole seminal microbiome from ecological perspective a routine research technique. For example, Weng et al. (2014) showed that the most abundant genera among the semen samples of 96 Chinese individuals were *Lactobacillus* (19.9%), *Pseudomonas* (9.85%), *Prevotella* (8.51%), and *Gardnerella* (4.21%). They further found that the seminal bacterial communities were clustered (through unsupervised clustering analysis) into three major types, dominated by *Lactobacillus, Pseudomonas*, and *Prevotella*, respectively. They also investigated the association between seminal microbial community and semen quality. In spite of the significant advances made in the existing studies, to the best of our knowledge, no studies with biogeography approaches to seminal fluid microbiome have ever been performed. As mentioned previously, biogeography approaches offer applicable theory and ideal techniques for analyzing the spatial distribution patterns of seminal microbiome diversity in a human population or cohort, and insights from the biogeography approaches such as heterogeneities of the seminal microbiome among individuals and the population-level characteristics should certainly be rather useful for personalized fertility research and public health.

Microbial biogeography is charged with the mission of understanding the spatial and/or temporal distribution of microbial diversities on regional or global scales. The classic species-area relationship (SAR), which quantitatively characterizes the relationship between the number of species (formally known as *species richness*, which is a rough measure of biodiversity) and the geographic area species distributed as a power-law function, is regarded as one of few classic laws in ecology and biogeography. The first documentation of the SAR relationship can be traced back to British botanist (Watson's, 1835) study of the distribution of plants. Since then, numerous theoretical and field studies have been performed (Watson, 1835; Preston, 1960; Connor and McCoy, 1979; Rosenzweig, 1995; Harte et al., 1999; Lomolino, 2000; Drakare et al., 2006; Tjørve and Tjørve, 2008; Tjørve, 2009; He and Hubbell, 2011; Sizling et al., 2011; Storch et al., 2012; Triantis et al., 2012; Whittaker and Triantis, 2012; Helmus et al., 2014). In the 1960's, the SAR theory inspired (MacArthur and Wilson's, 1967) establishment of their island biogeography theory, and the theory not only greatly enriched the principles and methods of general biogeography, but also was essential in shifting the focus of ecological research from population to community and in advancing *community ecology* in the 1970s and after. Today, much of the ecological theories and analysis techniques applied to microbiome research come from *community ecology*.

Recently, taking advantage of the big metagenomic datasets from the human microbiome project (HMP) and related studies, Ma, 2018a,b extended the classic SAR to general DAR (diversity-area relationship) by replacing the “species richness” in the classic SAR with general “diversity.” As mentioned previously, *species richness* or the number of species in a community, region or area, is rather rough as a measure of biodiversity because it ignores the fact that not all species are born equally abundant on the planet. Some species like panda are on one extreme and others such as flies are another extreme. The classic SAR is therefore somewhat flawed when the relationship is used to characterize the spatial distribution of biodiversity thanks to the simplified measure of biodiversity with species numbers. The DAR overcomes the flaw of traditional SAR by using more scientific metrics for biodiversity measures. Specifically, to construct DAR models, Ma, 2018a,b utilized Renyi's entropy based Hill numbers, which included some of the most widely used diversity indexes such as Shannon diversity and Simpson diversity indexes as its special cases. The adoption of Hill numbers for building DAR models also overcomes an issue of selecting diversity index from many of the choices, which often confuses non-ecologists unnecessarily.

The present article aims to apply the recent extended DAR modeling approach to discovering the important patterns of biogeography of seminal microbiome. We build the DAR models and compute these metrics by using the metagenomic sequencing data originally reported by Weng et al. (2014), and we also explore whether or not those metrics are related to the sperm quality. Specifically, we build DAR models for alpha-diversity and beta-diversity, respectively, and further derive some critical parameters including *diversity scaling parameter*—measuring the change rates of diversity across individuals (the size of microbial habitat area), *pair-wise diversity overlap* (similarity) (PDO)—measuring the average proportion of shared diversity or similarity between two individuals, *maximum accrual diversity* (MAD) in a population or cohort, and the ratio of individual to population diversity (*RIP*), a newly introduced metric that measures the ratio of individual microbial diversity to population-level microbial diversity. In terms of more general biogeography terms beyond the human microbiome, the concept of RIP can be generalized as the ratio of local to regional diversity (LRD) or ratio of local to global diversity (LGD), which can be applied to general biodiversity research in any other ecosystems.

## Materials and Methods

### Datasets Description

The 16S-rRNA OTU (operational taxonomic unit) tables of the semen microbiome at genus and species taxonomic levels, respectively, which we used to perform the DAR analysis, were originally reported by Weng et al. (2014). The OTU tables were generated from DNA-sequencing the semen microbiome samples, collected from 96 individuals including 35 with normal fertility, 28 with sub-normal fertility, and 33 with abnormal fertility, and the consequent bioinformatics analysis. From the 96 samples, Weng et al. (2014) obtained a total of 8,337,766 sequence reads, that is 80,424 reads per participant sample, a sufficiently large sample size for consequent statistical analyses. They detected an average number of 135 genera and 569 species from those samples.

Since the objective of Weng et al. (2014) study was to investigate the relationship between sperm quality and seminal microbiome, the original study included three treatments (groups), i.e., the normal, sub-normal, and abnormal as mentioned previously. The study design, of course, has no issue at all with its original objectives. To harness the data for our DAR analysis in this study, we first build DAR models for each treatment separately, and then perform statistical tests to see if there are any differences in the DAR parameters among the three treatments. If there is any significant difference, we keep the results and further investigate the implications of the difference to the status of treatments (fertility status). If there is not any significant difference, we then combine all 96 samples from the three treatments, build a single set of DAR models with the combined datasets, and further use the DAR models to explore the general biogeography properties of the seminal microbiome.

### The Diversity-Area Relationship (DAR)

The process of constructing DAR models for microbes consists of the following three steps: (i) bioinformatics analysis of 16S-rRNA reads to get OUT tables (Schloss et al., 2009; Caporaso et al., 2010; Bokulich et al., 2018); (ii) computing species or OTU diversities measured with the Hill numbers (Chao et al., 2012, 2014; Ma, 2017); (iii) building the DAR models (Ma, 2018a,b).

#### Diversity Measured in Hill Numbers

The Hill numbers are a form of Renyi's entropy (Renyi, 1961). It was initially introduced as an *evenness* index from economics by Hill (1973) and later reintroduced into ecology by Jost (2007) and Chao et al. (2012) who further clarified Hill's numbers for measuring alpha diversity as:


(1) $$\begin{array}{l} {\;}^{q}D={\left(\overset{S}{\underset{i=1}{\sum }}p_{i}^{q}\right)}^{1/\left(1-q\right)} \end{array}$$

where *S* is the number of species, *p*_i_ is the relative abundance of species *i, q* is the order number of diversity.

The Hill number is undefined when *q* = 1, but its limit as *q* approaches to *1* exists in the following form:


(2) $$\begin{array}{l} {\;}^{1}D={\underset{q\to 1}{\lim}}^{q}D=\exp\left(-\overset{s}{\underset{i=1}{\sum }}p_{i}\log\left(p_{1}\right)\right) \end{array}$$

The parameter *q* controls the sensitivity of the Hill number to the relative frequencies of species abundances. When *q* = 0, the species abundances do not weigh at all and ^*0*^*D* = *S*, i.e., species richness. When *q* = 1, ^*1*^*D* equal the *exponential* of Shannon entropy, and is interpreted as the number of typical or common species in the community because ^*1*^*D* is weighted proportionally by species abundances. When *q* = 2, ^2^*D* equal the reciprocal of Simpson index, i.e.,


(3) $$\begin{array}{l} {\;}^{2}D=\left(1/\overset{S}{\underset{i=1}{\sum }}p_{i}^{2}\right) \end{array}$$

which is interpreted as the number of *dominant* or very abundant species in the community (Chao et al., 2012) because ^2^*D* is weighted in favor of more abundant species. The general interpretation of ^*q*^*D* is that the community has a diversity of order *q*, which is equivalent to the diversity of a community with ^*q*^*D* = *x* equally abundant species.

A recent consensus suggested that, with the Hill numbers, the multiplicatively defined beta-diversity, rather than additively defined, by partitioning gamma diversity into the product of alpha and beta, should be used to define beta-diversity, in which both alpha and gamma diversities are measured with the Hill numbers.


(4) $$\begin{array}{l} {\;}^{q}D_{\beta }{=}^{q}D_{\gamma }{/}^{q}D_{\alpha } \end{array}$$

This beta diversity derived from the above partition takes the value of *1* if all communities are identical, the value of *N* (the number of communities) when all the communities are completely different from each other (there are no shared species). With Jost (2007) words, this beta diversity measures “*the effective number of completely distinct communities*.” In this study, we compute diversities until *q* = 3, i.e., to the third order. Note that a series of the Hill numbers at different order *q* is termed *diversity profile* (Jost, 2007; Chao et al., 2012, 2014).

#### The DAR (Diversity-Area Relationship) Models

Based on the fact that all Hill numbers are in the units of species or species equivalents such as OTUs, and on the intuition that Hill numbers should follow the same or similar pattern of the classic SAR (species area relationship), Ma (2018a) extended SAR to general DAR (diversity-area relationship), in which diversity is measured with Hill numbers.

The basic power function, known as the *power law* (PL) species scaling law widely adopted in SAR study, is extended to describe the general diversity-area relationship (DAR):


(5) $$\begin{array}{l} {\;}^{q}D=cA^{z} \end{array}$$

where ^*q*^*D* is diversity measured in the *q*-*th* order Hill numbers, *A* is *area*, and *c* and *z* are parameters.

A slightly modified PL model, the power law with exponential cutoff (PLEC) model, originally introduced to SAR modeling by Plotkin et al. (2000) and Ulrich and Buszko (2003), respectively (also see Tjørve, 2009), can also be utilized for DAR modeling. The PLEC model is:


(6) $$\begin{array}{l} {\;}^{q}D=cA^{z}\exp\left(dA\right), \end{array}$$

where *d* is a third parameter and should be negative in DAR scaling models, and exp(*dA*) is the exponential decay term that eventually overwhelms the power law behavior at very large value of *A*.

The following log-linear transformed equations (7, 8) can be used to estimate the model parameters of Equations (5, 6), respectively:


(7) $$\begin{array}{lll} \ln\;\left(D\right) & = & \ln\;\left(c\right)+z\ln\;\left(A\right) \end{array}$$

(8) $$\begin{array}{lll} \ln\;\left(D\right) & = & \ln\;\left(c\right)+z\ln\;\left(A\right)+dA \end{array}$$

Both linear correlation coefficient (*R*) and *p*-value can be used to judge the goodness of the model fitting. In fact, either of them should be sufficient to judge the suitability of the models to data. Three advantages are associated with the linear-transformed fitting: (i) simplicity in computation, (ii) parameter z is scale-invariant with Equation (7), (iii) the ecological interpretation of scaling parameter is preserved with Equation (8). The scaling parameter *z* is also termed the *slope* of the DAR power-law, because *z* represents the *slope* of the linearized function in log–log space.

The relationship between DAR model parameter (*z*) of the DAR PL model and the diversity order (*q*), or *z-q* trend, was defined as the *DAR profile* (Ma, 2018a). It describes the change of diversity scaling parameter (*z*) with the diversity order (*q*), comprehensively. Obviously, the DAR profile is an extension of the *diversity profile* concept Chao et al. (2012, 2014) proposed, which is the diversity in the Hill numbers at the *q*-th order.

In macro-ecology, there are usually natural spatial orders for the “areas,” which is generally lacking in human microbiome because human residences are little relevant to the accrual of diversities for DAR modeling. To avoid the potential bias from an arbitrary order of the human microbiome samples, we totally permutated the orders of all the microbiome samples under investigation, and then randomly chose 100 orders of the microbiome samples generated from the total permutations. That is, rather than taking a single arbitrary order for accruing microbiome samples in one-time fitting to the DAR model, we repeatedly perform the DAR model-fitting 100 times with the 100 randomly chosen permutation orders. Finally, the averages of the model parameters from the 100 times of DAR fittings are adopted as the model parameters of the DAR for the set of microbiome samples under investigation. An additional advantage of this re-sampling from total permutations is that the procedure makes the parameter *c* of the DAR-PL model being able to represent an average individual in the population (cohort) from which the individual comes from.

#### Predicting MAD (Maximal Accrual Diversity) With DAR-PLEC Models

Ma (2018a) derived the maximal accrual diversity (MAD) in a cohort (or population) based on the PLEC model [Equations (6, 8)] as follows:


(9) Max(Dq)=qDmax=c(−zd)zexp(−z)=cAmaxzexp(−z)

and the number of individuals (*A*_max_) needed to reach the maximum can be estimated by


(10) $$\begin{array}{l} A_{\max}=-z/d \end{array}$$

where all parameters are the same as Equations (6,8).

Similar to the previous definition for DAR profile (*z-q* pattern), the MAD profile (*D*_max_-*q* pattern), was defined as a series of *D*_max_ values corresponding to different diversity order (*q*) (Ma, 2018a).

#### Pair-Wise Diversity Overlap (PDO) Profile

The *pair-wise diversity overlap* (*g*) of two bordering areas of the same size (i.e., the proportion of the new diversity in the second area) is (Ma, 2018a):


(11) $$\begin{array}{l} g=2-2^{z} \end{array}$$

where *z* is the scaling parameter of DAR-PL model [Equations (5, 7)]. If *z* = 1, then *g* = 0, there is no overlap (similarity); if *z* = 0, then *g* = 1, totally overlap. In reality, *g* should be between 0 and 1.

Since the *equal size* of *area* assumption is largely true in the case of sampling human microbiome, the parameter *z* of the DAR-PL can be utilized to estimate the *pair-wise diversity overlap* (PDO), i.e., the diversity overlap (similarity) between two individuals, in the human microbiome with Equation (11).

Similar to previous definitions for DAR profile (*z-q* pattern) and MAD profile (*D*_max_-*q* pattern), the PDO profile (*g-q* pattern) was defined as a series of PDO-*g* values at different diversity order (*q*) (Ma, 2018a).

#### A Summary on the Interpretations of Important DAR Parameters

We summarize the ecological interpretations from PL/PLEC as follows to facilitate the discussion of the results from fitting DAR models with semen microbiome datasets.

*z*: The slope of the DAR-PL model or *scaling* parameter, and it is the ratio of diversity accrual rate to area increase rate. The *DAR profile* is a series of *z-q* values, corresponding to different diversity order (*q*).

*c*: Theoretically, by setting *A* = 1, *S*_0_ = *cA*^*z*^ = *c*, hence *c* is the Hill numbers (i.e., the number of species or species equivalents of diversity) in *one unit of area*, but not *per unit of area* because the scaling is non-linear. However, since we used 100 times of re-sampling to get the DAR parameters as explained previously, plus that the area size in human microbiome sampling can be considered as approximately equal, we argue that, in practice, the parameter *c* of the DAR-PL model can be treated as an estimate of the individual-level diversity in Hill numbers, or of the diversity of an averaged individual in the cohort (or population) he or she belongs to.

*g*: The pair-wise diversity overlap (PDO) parameter. It measures the pair-wise diversity similarity between two neighboring areas of the same size, i.e., between two individuals in a cohort (or population). The *PDO profile* is a series of *g-q* values, corresponding to different diversity order (*q*).

*D*_*max*_: The maximal accrual diversity (MAD) parameter. It estimates the maximal accrual diversity across individuals. Theoretically, it should be specific to the microbiome type (e.g., the gut microbiome or semen microbiome). The *MAD profile* is a series of *D*_*max*_*-q* values, corresponding to different diversity order (*q*).

#### RIP (the Ratio of Individual Diversity to Population Accrual Diversity)—A New Definition

We define the RIP (Ratio of Individual diversity to Population accrual diversity) as:


(12) $$\begin{array}{l} {\;}^{q}RIP{=}^{q}c{/}^{q}D \end{array}$$

where ^*q*^*c* is the DAR-PL parameter at diversity order of *q*, and ^*q*^*D* is the estimated accrual diversity of the population (cohort) with DAR-PL model at diversity order of *q*.

We further define ^*q*^*RIP-q* series (there is a *RIP* for each diversity order *q*) as RIP profile, similar to the previously defined DAR-, PDO-, and MAD-profiles.

According to the above RIP definition, a RIP profile can be constructed with population (cohort) of any size. However, in practice, using ^*q*^*D*_*max*_ in place of ^*q*^*D* should be more convenient, that is:


(13) $$\begin{array}{l} {\;}^{q}RIP{=}^{q}c{/}^{q}D_{\max} \end{array}$$

The RIP parameter measures the average level of an individual can represent a population (or cohort) from which the individual comes from. As argued previously, parameter *c* is an *approximated* value of individual diversity (or diversity *per* individual). The approximation is contingent on two implicit assumptions: (i) the sizes of areas are equal, which is generally true in the case of human microbiome; (ii) the start of area accrual won't exert significant influence on the estimation of parameter *c*. This appears to be satisfied given that assumption (i) is largely true for the human microbiome. However, given the well-known inter-individual heterogeneity of the human microbiome, the choice of starting area (individual) to accrue diversity may indeed have a significant impact on the estimate of parameter *c*. To deal with the issue associated with assumption (ii), we adopt the previously introduced the re-sampling approach from total permutations of the microbiome samples, and use the average parameters from certain times (usually 100 should be enough) of repeatedly DAR model-fitting from the re-sampling.

In general biogeography terms beyond human microbiome, the previous definitions for RIP can be generalized as LRD (ratio of local to regional diversity) (Equation 14) or as LGD (ratio of local to global diversity (Equation 15). Both can be applied to measure the relationship between the local and regional/global biodiversities in any ecosystems. LRD & LGD are defined as:


(14) $$\begin{array}{lll} {\;}^{q}LRD & = & {\;}^{q}c{/}^{q}D \end{array}$$

(15) $$\begin{array}{lll} {\;}^{q}LGD & = & {\;}^{q}c{/}^{q}D_{\max} \end{array}$$

where the symbols (parameters) in the right have the same interpretations as in Equations (13,14).

## Results and Discussion

### Test the Differences in Semen DAR Parameters Among the Three Groups

We aimed to test whether or not there are significant differences among the three groups (normal, sub-normal and abnormal) in their DAR parameters. To perform this test, we built DAR models (including both alpha-DAR and beta-DAR models) for each group separately and then performed the randomization tests for the parameters of those DAR models. The parameters of the alpha-DAR models and beta-DAR models for the three different groups were listed in Tables S1, S2 of the online supplementary information (OSI), respectively. The results from the randomization test for the model parameters were listed in Table S3 (for alpha-DAR parameters) and Table S4 (for beta-DAR parameters), respectively. It turned out that there were no significant differences in any of the major DAR parameters between the groups, as revealed by the *p*-values (*p* > 0.05) in the last column of Tables S3, S4.

### Biogeography Analysis of the Semen Microbiome With DAR Modeling

#### Alpha-DAR Modeling

**Figure 1:**
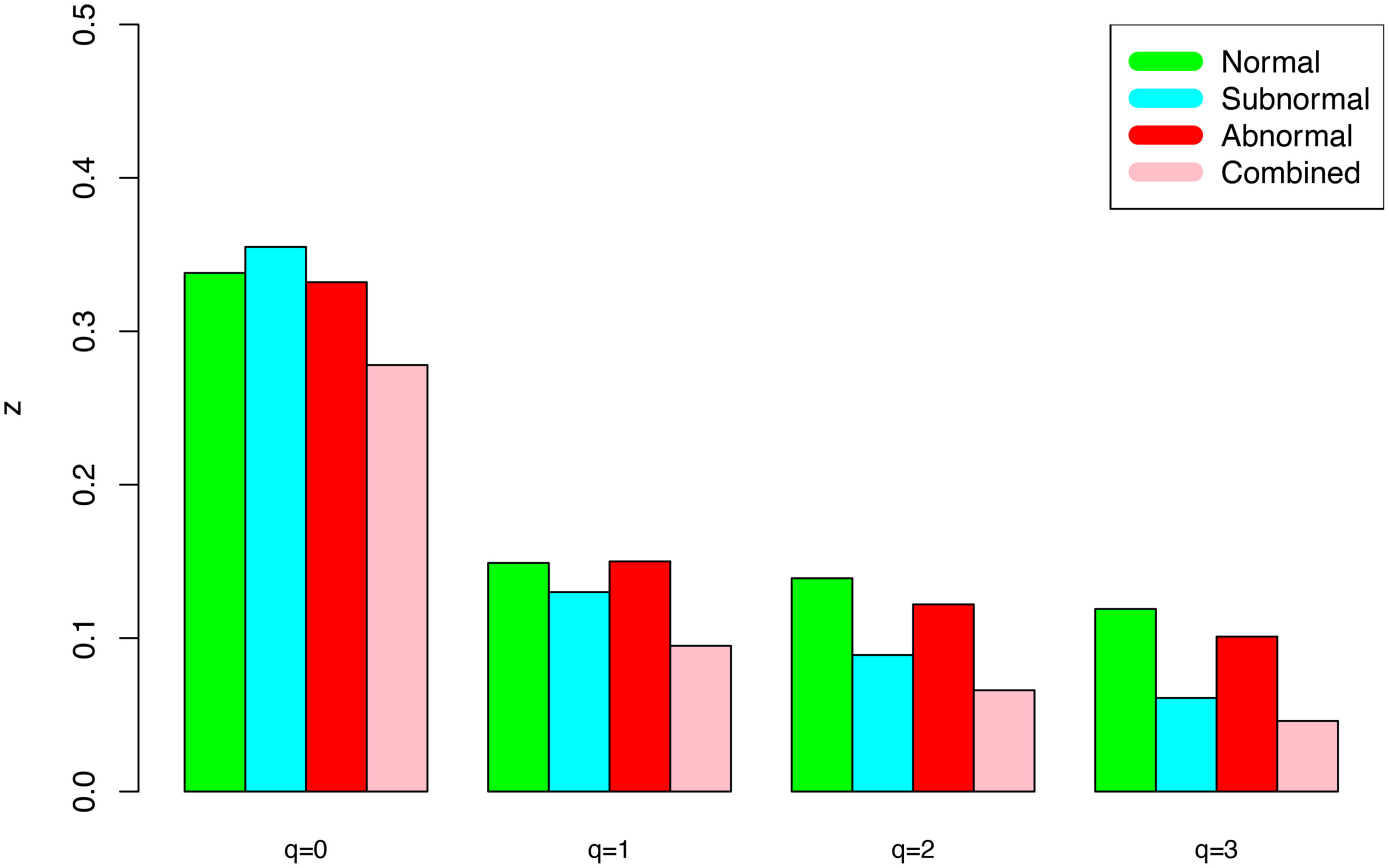
The alpha-DAR profile scaling parameter (*z-q* series) for the semen microbiome alpha-diversity at the genus level, for normal, sub-normal, abnormal, and combined groups.

**Figure 2:**
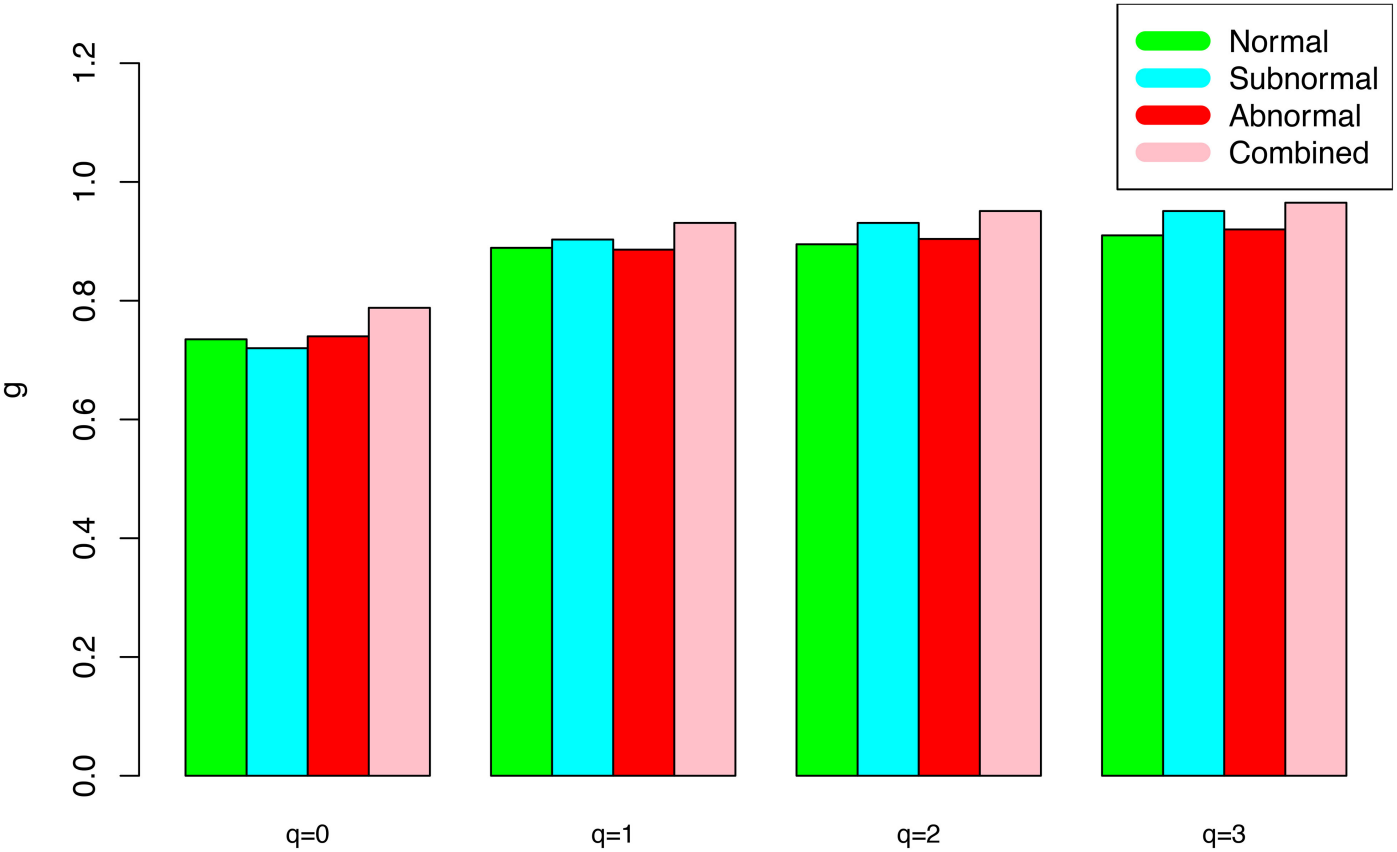
The alpha-PDO profile (*g-q* series) for the semen microbiome *alpha*-diversity at the genus level for normal, sub-normal, abnormal, and combined groups, respectively.

**Figure 3:**
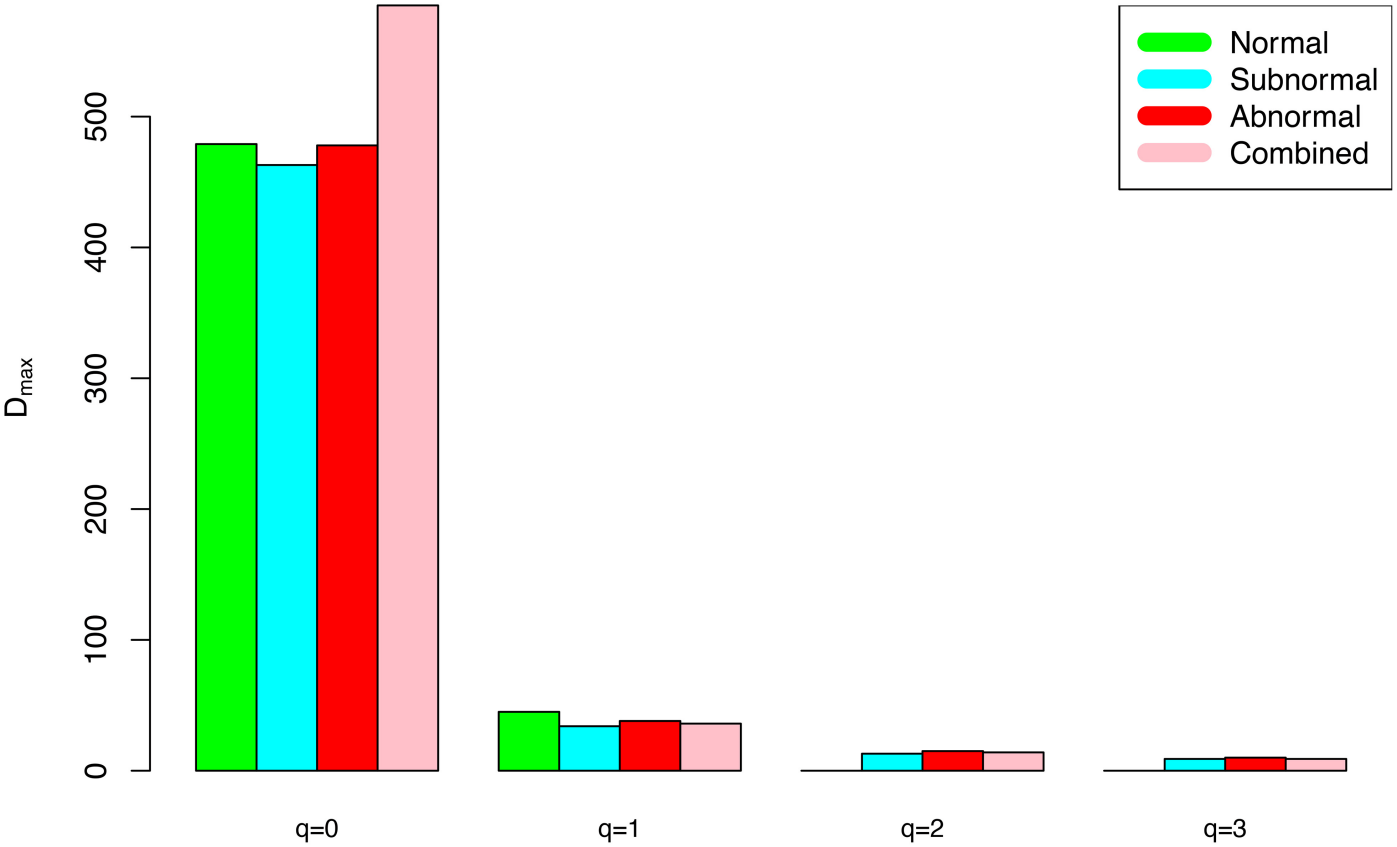
The alpha-MAD profile (*D*_*max*_-*q* series) for the semen microbiome *alpha*-diversity at the genus level, for normal, sub-normal, abnormal, and combined groups, respectively.

**Figure 4:**
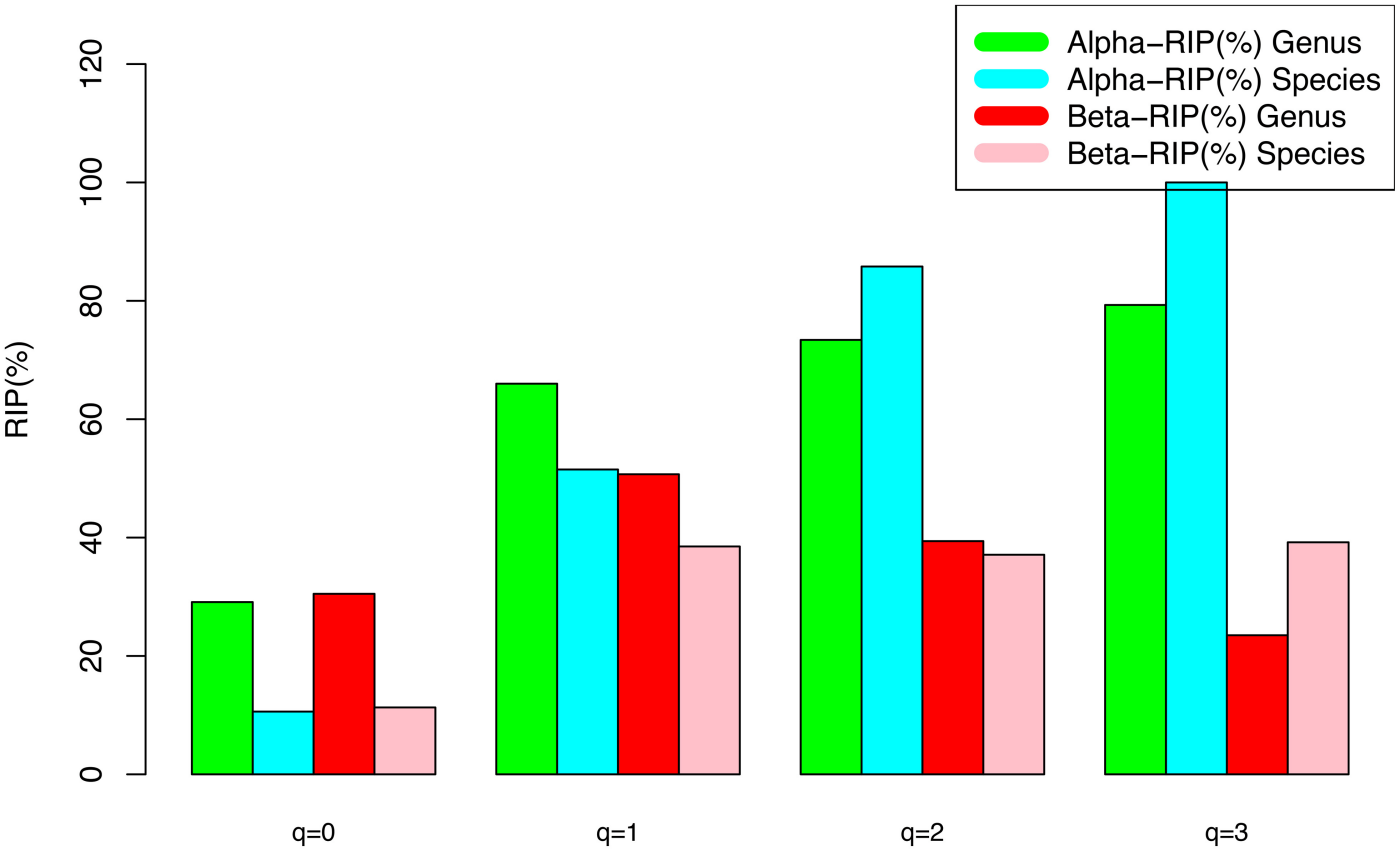
The RIP-profile (RIP-*q* series) for the semen microbiome diversity (alpha and beta diversity, respectively) at the genus level, for the normal, sub-normal, abnormal, and combined groups, respectively.

Tables 1, 2 listed the alpha-DAR parameters for the human semen microbiome at the genus and species level, respectively. The leftmost column in both the tables listed the diversity order (*q* = 0, 1, 2, 3) and, and the parameters for DAR-PL models and DAR-PLEC models were listed in the left and right side, respectively. From Tables 1, 2, we summarize the following findings:

- The DAR models fitted to the semen microbiome diversity in the Hill numbers at both genus and species levels statistically significant (*p* < 0.05 in 6 cases and *p* < 0.1 in two cases). Judged from the success rates among 100 times of random re-sampling, the PLEC model performed slightly better than the PL model, and species-level modeling slightly better than genus level. Therefore, the PLEC model at the species level performed best among four categories of the models.
- At both genus and species levels, the DAR scaling parameter *z* decreased monotonically with diversity order *q*, and the species level parameters are generally larger than their genus level counterparts. In the case of scaling parameter *z*, larger *z*-value indicates larger PL *slope* or fast change rates of diversity per unit accrual of area. This result should be expected obviously because the differences among individual subjects should be smaller at higher taxonomic level (genus) than lower level (species). In other words, the resolution of higher (genus) taxonomic level is rougher than that of the lower (species) taxonomic level. Figure 1 exemplified the DAR profiles of the alpha-diversity at the genus level, for the normal, sub-normal, abnormal, and combined groups, respectively.
- At both genus and species levels, the PDO (pair-wise diversity overlap) parameter (*g*) showed a monotonically increasing trend, which is opposite with that of the scaling parameter (*z*) as expected. The PDO parameter confirmed the previous finding that semen microbiome has higher similarity (overlap) at genus level than at species level, indicated by higher *g*-value. Figure 2 exemplified the PDO profiles of the alpha-diversity at the genus level, for the normal, sub-normal, abnormal, and combined groups, respectively.
- The negative *d*-values of all PLEC models at both genus and species levels, indicated the existence of asymptote lines and the necessity of introducing more sophisticated PLEC model, which also made the prediction of MAD (maximal accrual diversity) or *D*_*max*_ possible. The MAD (*D*_*max*_) decreased with the increase of diversity order (*q*), as determined by the nature (definition) of the Hill numbers. The MAD at *q* = 0, or ^*0*^*D*_*max*_ which is simply the maximal accrual of microbial species (genus) *richness* of the population of individuals. Figure 3 exemplified the MAD profiles of the alpha-diversity at the genus level, for the normal, sub-normal, abnormal, and combined groups, respectively.
- **Table 5** further computed the RIP [Ratio of Individual diversity to Population maximal accrual diversity: Equation (12b)] for all DAR models listed in Tables 1–**4**. The left side is the RIP computed from alpha-DAR parameters, and the right side is that computed from beta-DAR parameters. The RIP parameter measures the average level of an individual can represent a population from which he or she comes from. For example, at diversity order *q* = 0, i.e., species (genus) richness level, the alpha-diversity of an individual, on *average*, contains approximately 10.6% (species level) or 29.1% (genus-level) of the diversity accrued by the population. When the diversity order (*q*) increases, the RIP percentage is also increased, as indicated by **Table 5**. Note that since RIP is defined in terms of an *averaged* individual, it may be a poor representative for a specific individual, especially when the inter-subject heterogeneity of diversity is high. Figure 4 exemplified the RIP profiles of the alpha-diversity at the genus level, for the normal, sub-normal, abnormal, and combined groups, respectively.

**Table 1:** The parameters of *alpha*-DAR (*alpha*-diversity-area relationship) computed with 100 times of re-sampling at **genus** level for the human semen microbiome.

**Diversity order and statistics**		**Power law (PL)**						**PL with exponential cutoff (PLEC)**							
		***z***	**ln(*c*)**	***R***	***p*-value**	***g***	***N*** ^*^	***z***	***d***	**ln(*c*)**	***R***	***p*-value**	***N***	***A_***max***_***	***D_***max***_***
*q* = 0	Mean	0.277	5.148	0.984	0.000	0.788	100	0.344	−0.002	5.027	0.993	0.000	100	139	590.8
	Std. Err.	0.031	0.129	0.012	0.000	0.026		0.065	0.002	0.181	0.006	0.000			
	Min	0.211	4.812	0.936	0.000	0.718		0.218	−0.006	4.533	0.963	0.000			
	Max	0.359	5.444	0.998	0.000	0.843		0.514	0.000	5.384	0.999	0.000			
*q* = 1	Mean	0.100	3.176	0.668	0.039	0.927	94	0.154	−0.002	3.078	0.761	0.022	95	77	36.3
	Std. Err.	0.076	0.318	0.250	0.168	0.056		0.131	0.003	0.377	0.215	0.112			
	Min	−0.049	2.303	0.008	0.000	0.774		−0.230	−0.010	2.005	0.061	0.000			
	Max	0.294	3.832	0.975	0.940	1.034		0.546	0.010	4.051	0.987	0.839			
*q* = 2	Mean	0.075	2.308	0.514	0.061	0.944	89	0.125	−0.002	2.217	0.667	0.013	97	67	13.7
	Std. Err.	0.107	0.463	0.250	0.208	0.080		0.170	0.005	0.490	0.200	0.084			
	Min	−0.133	1.013	0.003	0.000	0.721		−0.365	−0.011	0.723	0.069	0.000			
	Max	0.355	3.171	0.937	0.976	1.088		0.639	0.017	3.342	0.968	0.799			
*q* = 3	Mean	0.055	1.998	0.455	0.093	0.958	76	0.102	−0.002	1.914	0.629	0.030	92	59	9.3
	Std. Err.	0.111	0.483	0.268	0.208	0.082		0.172	0.005	0.489	0.216	0.123			
	Min	−0.161	0.719	0.000	0.000	0.744		−0.385	−0.012	0.439	0.073	0.000			
	Max	0.329	2.877	0.942	0.999	1.105		0.602	0.017	2.943	0.959	0.780			

*N*

*, *the number of successful fitting to DAR model from 100 times of random re-sampling of the individual orders*.

**Table 2:** The parameters of *alpha*-DAR (*alpha*-diversity-area relationship) computed with 100 times of re-sampling at **species** level for the human semen microbiome.

**Diversity order and statistics**		**Power law (PL)**						**PL with exponential cutoff (PLEC)**							
		***z***	**ln(*c*)**	***R***	***p*-value**	***g***	***N*** ^*^	***z***	***d***	**ln(*c*)**	***R***	***p*-value**	***N***	***A_***max***_***	***D_***max***_***
*q = 0*	Mean	0.507	6.627	0.984	0.000	0.577	100	0.637	−0.005	6.393	0.993	0.000	100	132	7099.5
	Std. Err.	0.061	0.247	0.012	0.000	0.060		0.127	0.003	0.354	0.006	0.000			
	Min	0.341	5.937	0.937	0.000	0.400		0.334	−0.012	5.357	0.962	0.000			
	Max	0.678	7.347	0.998	0.000	0.734		1.000	0.002	7.213	0.999	0.000			
*q = 1*	Mean	0.158	4.570	0.681	0.019	0.881	93	0.231	−0.003	4.438	0.790	0.004	98	85	187.6
	Std. Err.	0.115	0.491	0.234	0.084	0.091		0.215	0.006	0.590	0.185	0.037			
	Min	−0.156	3.180	0.068	0.000	0.613		−0.343	−0.020	2.785	0.147	0.000			
	Max	0.472	5.949	0.983	0.508	1.102		0.917	0.013	5.817	0.988	0.362			
*q = 2*	Mean	0.035	3.313	0.417	0.124	0.969	73	0.079	−0.002	3.235	0.663	0.005	96	49	32.0
	Std. Err.	0.157	0.683	0.273	0.239	0.116		0.306	0.009	0.770	0.196	0.022			
	Min	−0.322	1.548	0.015	0.000	0.645		−0.786	−0.022	1.217	0.208	0.000			
	Max	0.439	4.965	0.951	0.888	1.200		0.902	0.024	4.866	0.959	0.129			
*q = 3*	Mean	−0.022	2.893	0.453	0.072	1.009	81	0.010	−0.001	2.836	0.667	0.008	98	8	17.3
	Std. Err.	0.158	0.693	0.258	0.181	0.112		0.306	0.010	0.762	0.191	0.062			
	Min	−0.388	1.144	0.020	0.000	0.690		−0.803	−0.024	0.945	0.101	0.000			
	Max	0.389	4.489	0.944	0.846	1.236		0.768	0.023	4.422	0.948	0.619			

*N*

*, *the number of successful fitting to DAR model from 100 times of random re-sampling of the individual orders*.

#### Beta-DAR Modeling

Tables 3, 4 listed the beta-DAR parameters for the human semen microbiome at the genus and species level, respectively. The leftmost column in both the tables are the diversity order (*q* = 0, 1, 2, 3) and, and the parameters for beta-DAR PL models and beta-DAR PLEC models were listed in the left and right side, respectively. From both the tables, we observed the following findings:

- The beta-DAR models fitted to the semen microbiome beta-diversity data at both genus and species levels statistically significant (*p* < 0.05 in 7 cases and *p* < 0.1 in 1 case). Judged from the success rates among 100 times of random re-sampling, the beta-PLEC model performed slightly better than beta-PL model, and species-level modeling slightly better than the genus-level. Therefore, the beta-PLEC model at the species level performed best among four categories of the models.
- At both genus and species levels, the beta-DAR scaling parameter *z* exhibited a valley-shaped pattern with diversity order (*q*), and the species level parameters are generally larger than their genus level counterparts. In the case of scaling parameter *z*, larger z-value indicates larger slope or faster change rates of diversity per unit change of area accrual. This result should be expected obviously because the differences among individual subjects should be smaller at higher taxonomic level (genus) than lower level (species).
- At both genus and species levels, the beta-PDO parameter (*g*) showed a mountain-shaped trend, which is opposite with that of the scaling parameter (*z*) as expected. The beta-PDO parameter confirmed previous finding that semen microbiome has higher level of similarity at genus level than at species level, indicated by higher *g*-value, which measures the pair-wise diversity overlap (similarity).
- The negative *d*-values of all beta-PLEC models at both genus and species levels, indicated the existence of asymptote lines and the necessity for introducing the more sophisticated beta-PLEC model, which also made the prediction of beta-*D*_*max*_ (beta-MAD) possible. The beta-MAD-*q* or beta-*D*_*max*_-*q*, or beta-MAD profile, exhibited a valley-shaped trend, which is consistent with the z-q series or DAR profile. The beta-MAD or at *q* = 0, ^*0*^*D*_*max*_, which is simply the species (genus) *richness* or the number of species (genus) in the cohort (or population).
- Table 5 listed the RIP profile, i.e., the ratio of individual diversity to population maximal accrual diversity (Equation 12b), at different diversity order (*q*), for all DAR models listed in Tables 1–4. The two right columns were computed for beta-DAR models from Tables 3, 4 (the left side for alpha-DAR). The RIP parameter (profile) measures the average level of an individual can represent a population from which he or she comes from. For example, when q = 0 (species or genus richness level), beta-*RIP* = 11.3% at species level, and beta-*RIP* = 30.5% at genus level. This suggested that beta-diversity of an individual, on average, contains ~11.3% (at the species level) and 30.5% (at the genus level) of the diversity accrued at the population level. When the diversity order (*q*) increases, the RIP also increased accordingly.

**Table 3:** The parameters of *beta*-DAR (*beta*-diversity area relationship) computed with 100 times of re-sampling at **genus** level.

**Diversity order & statistics**		**Power law (PL)**						**PL with exponential cutoff (PLEC)**							
		***z***	**ln(*c*)**	***R***	***p*-value**	***g***	***N*** ^*^	***z***	***d***	**ln(*c*)**	***R***	***p*-value**	***N***	***A_***max***_***	***D_***max***_***
*q* = 0	Mean	0.266	0.294	0.990	0.000	0.797	100	0.338	−0.002	0.152	0.997	0.000	100	140	4.4
	Std. Err.	0.014	0.059	0.005	0.000	0.012		0.025	0.001	0.067	0.002	0.000			
	Min	0.235	0.160	0.975	0.000	0.773		0.272	−0.004	0.023	0.988	0.000			
	Max	0.296	0.424	0.999	0.000	0.823		0.395	−0.001	0.341	0.999	0.000			
*q* = 1	Mean	0.160	0.483	0.800	0.004	0.882	99	0.277	−0.004	0.252	0.895	0.000	100	70	3.2
	Std. Err.	0.061	0.273	0.165	0.045	0.047		0.107	0.004	0.255	0.092	0.000			
	Min	−0.010	−0.103	0.079	0.000	0.772		0.010	−0.012	−0.339	0.483	0.000			
	Max	0.296	1.225	0.982	0.448	1.007		0.556	0.005	0.943	0.984	0.000			
*q* = 2	Mean	0.225	0.595	0.782	0.008	0.829	99	0.393	−0.006	0.264	0.872	0.000	100	69	4.6
	Std. Err.	0.084	0.364	0.166	0.083	0.068		0.171	0.005	0.401	0.107	0.000			
	Min	−0.005	−0.321	0.022	0.000	0.623		−0.066	−0.018	−0.886	0.470	0.000			
	Max	0.462	1.564	0.977	0.829	1.003		0.864	0.009	1.112	0.983	0.000			
*q* = 3	Mean	0.273	0.586	0.804	0.002	0.789	99	0.441	−0.006	0.252	0.874	0.000	100	77	5.6
	Std. Err.	0.092	0.385	0.143	0.015	0.077		0.207	0.006	0.463	0.106	0.000			
	Min	0.033	−0.430	0.150	0.000	0.541		−0.070	−0.022	−1.080	0.509	0.000			
	Max	0.545	1.541	0.980	0.148	0.977		1.016	0.011	1.178	0.985	0.000			

*N*

*, *the number of successful fitting to DAR model from 100 times of random re–sampling of the individual orders*.

**Table 4:** The parameters of *beta*-DAR (*beta*-diversity area relationship) computed with 100 times of re-sampling at **species** level.

**Diversity order & statistics**		**Power law (PL)**						**PL with exponential cutoff (PLEC)**							
		***z***	**ln(*c*)**	***R***	***p*-value**	***g***	***N*** ^*^	***z***	***d***	**ln(*c*)**	***R***	***p*-value**	***N***	***A_***max***_***	***D_***max***_***
*q* = 0	Mean	0.480	0.389	0.994	0.000	0.605	100	0.588	−0.004	0.174	0.999	0.000	100	161	13.1
	Std. Err.	0.012	0.052	0.003	0.000	0.011		0.028	0.001	0.064	0.001	0.000			
	Min	0.453	0.222	0.985	0.000	0.568		0.534	−0.006	0.003	0.996	0.000			
	Max	0.518	0.506	0.998	0.000	0.631		0.666	−0.002	0.323	1.000	0.000			
*q* = 1	Mean	0.226	0.572	0.884	0.000	0.830	100	0.366	−0.005	0.294	0.944	0.000	100	77	4.6
	Std. Err.	0.051	0.236	0.090	0.000	0.041		0.107	0.004	0.230	0.042	0.000			
	Min	0.090	0.004	0.577	0.000	0.736		0.086	−0.016	−0.241	0.743	0.000			
	Max	0.338	1.205	0.983	0.000	0.935		0.670	0.005	0.816	0.989	0.000			
*q* = 2	Mean	0.235	0.732	0.781	0.000	0.821	100	0.405	−0.006	0.396	0.876	0.000	100	71	5.6
	Std. Err.	0.083	0.377	0.146	0.000	0.068		0.173	0.006	0.360	0.084	0.000			
	Min	0.074	−0.121	0.358	0.000	0.648		−0.028	−0.024	−0.392	0.623	0.000			
	Max	0.435	1.452	0.961	0.000	0.947		0.855	0.011	1.099	0.979	0.000			
*q* = 3	Mean	0.276	0.676	0.765	0.000	0.786	100	0.378	−0.003	0.474	0.838	0.000	100	109	6.5
	Std. Err.	0.103	0.438	0.143	0.002	0.088		0.228	0.008	0.430	0.124	0.001			
	Min	0.057	−0.294	0.248	0.000	0.574		−0.181	−0.024	−0.602	0.317	0.000			
	Max	0.512	1.478	0.945	0.016	0.959		0.868	0.019	1.353	0.980	0.008			

*N*

*, *the number of successful fitting to DAR model from 100 times of random re-sampling of the individual orders*.

An interesting observation is that alpha-RIP profile and beta-RIP profile exhibited different patterns: the former is monotonically increasing, but the latter is mountain-shaped. This pattern is clear from comparing of the left side and right side of Table 5.

**Table 5:** RIP (ratio of individual diversity to population maximal accrual diversity).

**Diversity order (*q*)**	**Alpha-RIP (%)**		**Beta-RIP (%)**	
	**Genus-level**	**Species-level**	**Genus-level**	**Species-level**
*q* = 0	29.1	10.6	30.5	11.3
*q* = 1	66.0	51.5	50.7	38.5
*q* = 2	73.4	85.8	39.4	37.1
*q* = 3	79.3	100	23.5	39.2

## Discussion

The results of DAR analysis presented above revealed that fertility status (normal, subnormal, abnormal) did not have a significant influence on biogeography of semen microbiome, specifically, on the inter-subject (spatial) heterogeneity in terms of either alpha-diversity or beta-diversity. Previous studies have suggested changes in semen microbiome diversity associated with fertility health (Hou et al., 2013; Weng et al., 2014), although no rigorous statistical tests were performed with the published studies. Furthermore, the diversity of a microbiome sample *per se* and the diversity scaling (or spatial heterogeneity changes, a topic of this study) within a population are very different concepts. Logically, the change of individual diversity does not necessary lead to changes of the diversity heterogeneity among individuals. Therefore, the lack of differences in the diversity scaling parameter (z) and other DAR parameters, among three groups with different fertility status do not contradict the published studies on the human semen microbiome.

The lack of significant differences among various fertility groups actually simplified our study, enabled us to build the DAR models for a general Chinese population. Using the DAR models, we were able to (i) estimate the diversity changes of semen microbiome in a human cohort (population) or DAR profile; (ii) predict the maximal accrual diversity (MAD) of semen microbiome in a human cohort (population) or the MAD profile; (iii) estimate the PDO (pair-wise diversity overlap or similarity) between two individuals or the PDO profile; (iv) assess the RIP profile (i.e., the ratio of individual diversity to population accrual diversity), which measures the level an individual can represent a population which he belongs to. The “*profiles*” provide series of key parameter associated with different diversity order (*q*), which weights diversity differently: from species richness (*q* = 0), where all species are weighted equally, to *q* = 3, where dominant species were weighted for more and rare species were weighted for less. These parameters sketched out the biogeography “maps” of the human semen microbiome in terms of the four profiles: the DAR-, PDO-, MAD-, and RIP profiles. Together, the four profiles (maps) comprehensively sketched out the biogeography of semen microbiome—the spatial distribution or inter-subject heterogeneity of semen microbiome diversity at different diversity orders (*q*). The different biogeography maps are similar to different geography maps, each may with different utilization (e.g., rainfall map vs. biodiversity map, both of different utilizations). Using another analogy, maps at different diversity order (*q*) are similar to the maps with different scales or resolutions.

Hence, similar to the obvious significance of geographic maps, our biogeographic maps for the human semen microbiome diversity distribution should be rather important for further investigating the spatial distribution (or inter-subject heterogeneity) of the semen microbiome and their biomedical implications. A limitation of this study is that the datasets we used were limited to a Chinese population. We hope that future studies will include datasets from other ethnic groups.

## Author Contributions

ZM designed the study, interpreted the results and wrote the paper. LL performed the computation and participated the interpretation of the results.

### Conflict of Interest Statement

The authors declare that the research was conducted in the absence of any commercial or financial relationships that could be construed as a potential conflict of interest.
