# Supplementary material for: Semen Microbiome Biogeography: An Analysis Based on a Chinese Population Study
Source: Front Microbiol. 2019 Jan 31;9:3333. doi: 10.3389/fmicb.2018.03333 (PMC6371047; doi:10.3389/fmicb.2018.03333)
Supplement: Supplementary file 1 [file Data_Sheet_1.PDF]

Online Supplementary Information for: **Semen Microbiome Biogeography Analysis: Exemplified with Chinese Population Samples**

Table S1. Fitting the *Alpha*-Diversity Area Relationship (*A*-DAR) model with the semen microbiome OTU diversity at the genus level

Datasets	Diversity Order & Statistics		Power Law (PL)						PL with Exponential Cutoff (PLEC)							
			z	ln(c)	R	p-value	g	N	z	d	ln(c)	R	p-value	N	A_{max}	D_{max}
Normal	*q=0*	Mean	0.338	4.980	0.985	0.000	0.735	35	0.417	-0.007	4.902	0.993	0.000	35	57	479
		Std. Err.	0.056	0.179	0.013	0.000	0.050		0.111	0.006	0.221	0.005	0.000			
		Min	0.229	4.392	0.934	0.000	0.570		0.212	-0.026	4.192	0.964	0.000			
		Max	0.516	5.330	0.998	0.000	0.828		0.783	0.006	5.304	0.999	0.000			
	q=1	Mean	0.149	2.982	0.701	0.067	0.889	35	0.152	0.000	2.979	0.810	0.014	35	556	45
		Std. Err.	0.096	0.299	0.271	0.200	0.074		0.216	0.013	0.392	0.174	0.091			
		Min	-0.041	2.247	0.017	0.000	0.688		-0.374	-0.024	2.077	0.139	0.000			
		Max	0.392	3.591	0.980	0.923	1.028		0.565	0.032	3.774	0.990	0.731			
	q=2	Mean	0.139	2.058	0.554	0.116	0.895	35	0.104	0.003	2.092	0.701	0.033	35	0	NA
		Std. Err.	0.121	0.389	0.277	0.268	0.094		0.293	0.021	0.478	0.208	0.109			
		Min	-0.112	1.037	0.005	0.000	0.645		-0.775	-0.035	1.010	0.144	0.000			
		Max	0.438	2.710	0.946	0.978	1.075		0.696	0.061	3.240	0.965	0.716			
	q=3	Mean	0.119	1.746	0.494	0.115	0.910	35	0.084	0.003	1.780	0.662	0.048	35	0	NA
		Std. Err.	0.121	0.395	0.263	0.229	0.093		0.300	0.023	0.469	0.215	0.143			
		Min	-0.137	0.740	0.009	0.000	0.665		-0.831	-0.036	0.722	0.102	0.000			
		Max	0.417	2.441	0.931	0.959	1.090		0.674	0.064	2.949	0.947	0.847			
Subnormal	*q=0*	Mean	0.355	4.985	0.983	0.000	0.720	28	0.454	-0.011	4.904	0.992	0.000	28	41	463
		Std. Err.	0.053	0.153	0.013	0.000	0.047		0.118	0.009	0.193	0.005	0.000			
		Min	0.226	4.568	0.940	0.000	0.582		0.216	-0.033	4.392	0.967	0.000			
		Max	0.503	5.364	0.998	0.000	0.831		0.741	0.005	5.372	0.999	0.000			
	q=1	Mean	0.130	3.136	0.688	0.047	0.903	28	0.219	-0.010	3.064	0.797	0.020	28	22	34
		Std. Err.	0.102	0.319	0.252	0.144	0.078		0.199	0.017	0.349	0.190	0.067			
		Min	-0.076	2.365	0.058	0.000	0.686		-0.243	-0.059	2.136	0.286	0.000			
		Max	0.394	3.804	0.992	0.769	1.051		0.708	0.028	3.787	0.993	0.345			
	q=2	Mean	0.089	2.280	0.500	0.148	0.931	28	0.190	-0.011	2.199	0.685	0.066	28	17	13
		Std. Err.	0.142	0.451	0.275	0.257	0.108		0.278	0.026	0.463	0.234	0.183			
		Min	-0.210	1.028	0.015	0.000	0.604		-0.458	-0.082	0.959	0.061	0.000			
		Max	0.481	3.077	0.958	0.941	1.135		0.921	0.071	2.998	0.978	0.954			
	q=3	Mean	0.061	1.982	0.439	0.185	0.951	28	0.177	-0.013	1.888	0.664	0.080	28	14	9
		Std. Err.	0.144	0.462	0.266	0.278	0.108		0.293	0.029	0.458	0.237	0.203			
		Min	-0.238	0.727	0.011	0.000	0.643		-0.504	-0.087	0.731	0.036	0.000			
		Max	0.440	2.734	0.951	0.957	1.152		0.957	0.074	2.636	0.965	0.984			
Abnormal	*q=0*	Mean	0.332	5.047	0.978	0.000	0.740	33	0.429	-0.009	4.957	0.989	0.000	33	46	478
		Std. Err.	0.059	0.182	0.018	0.000	0.051		0.124	0.008	0.229	0.009	0.000			
		Min	0.200	4.662	0.919	0.000	0.622		0.186	-0.025	4.494	0.950	0.000			
		Max	0.463	5.457	0.996	0.000	0.851		0.642	0.007	5.471	0.998	0.000			
	q=1	Mean	0.150	3.152	0.640	0.075	0.886	33	0.236	-0.008	3.072	0.767	0.029	33	28	38
		Std. Err.	0.132	0.427	0.255	0.211	0.105		0.245	0.017	0.466	0.193	0.127			
		Min	-0.132	1.648	0.008	0.000	0.499		-0.323	-0.048	1.632	0.115	0.000			
		Max	0.586	4.033	0.991	0.964	1.087		0.786	0.049	4.029	0.991	0.818			
	q=2	Mean	0.122	2.335	0.501	0.121	0.904	33	0.195	-0.007	2.267	0.686	0.039	33	27	15
		Std. Err.	0.167	0.551	0.271	0.232	0.132		0.327	0.027	0.563	0.212	0.120			
		Min	-0.194	0.553	0.002	0.000	0.454		-0.546	-0.065	0.623	0.142	0.000			
		Max	0.628	3.237	0.979	0.990	1.126		1.033	0.073	3.326	0.980	0.735			
	q=3	Mean	0.101	2.031	0.457	0.183	0.920	33	0.171	-0.007	1.966	0.665	0.057	33	25	10
		Std. Err.	0.164	0.545	0.284	0.292	0.127		0.337	0.029	0.547	0.221	0.152			
		Min	-0.186	0.356	0.002	0.000	0.513		-0.568	-0.066	0.447	0.081	0.000			
		Max	0.573	2.844	0.972	0.992	1.121		1.039	0.073	2.922	0.975	0.905			
	q=0	Mean	0.278	5.149	0.983	0.000	0.788	96	0.350	-0.003	5.018	0.993	0.000	96	130	585

Combined		Std. Err.	0.027	0.116	0.012	0.000	0.023		0.056	0.001	0.154	0.006	0.000			
		Min	0.217	4.844	0.938	0.000	0.730		0.212	-0.006	4.674	0.959	0.000			
		Max	0.345	5.420	0.997	0.000	0.837		0.483	0.001	5.359	0.999	0.000			
	q=1	Mean	0.095	3.199	0.652	0.026	0.931	96	0.157	-0.002	3.087	0.776	0.004	96	68	36
		Std. Err.	0.069	0.295	0.243	0.104	0.052		0.122	0.003	0.341	0.168	0.028			
		Min	-0.053	2.493	0.023	0.000	0.794		-0.143	-0.010	2.216	0.174	0.000			
		Max	0.271	3.830	0.969	0.824	1.036		0.436	0.007	3.822	0.979	0.240			
	q=2	Mean	0.066	2.346	0.448	0.149	0.951	96	0.131	-0.002	2.229	0.660	0.039	96	54	14
		Std. Err.	0.102	0.442	0.292	0.283	0.076		0.163	0.005	0.456	0.213	0.165			
		Min	-0.108	1.232	0.002	0.000	0.729		-0.317	-0.011	1.042	0.018	0.000			
		Max	0.346	3.155	0.938	0.983	1.072		0.470	0.012	3.139	0.950	0.985			
	q=3	Mean	0.046	2.038	0.446	0.090	0.965	96	0.108	-0.002	1.925	0.649	0.024	96	47	9
		Std. Err.	0.107	0.468	0.261	0.208	0.079		0.169	0.006	0.464	0.202	0.100			
		Min	-0.144	0.895	0.008	0.000	0.736		-0.353	-0.013	0.730	0.091	0.000			
		Max	0.338	2.955	0.928	0.940	1.095		0.444	0.013	2.842	0.938	0.679			

Table S2. Fitting the *Beta*-Diversity Area Relationship (*B*-DAR) model with the semen microbiome OTU diversity at the genus level

Datasets	Diversity Order & Statistics		Power Law (PL)						PL with Exponential Cutoff (PLEC)							
			z	ln(*c*)	*R*	*p*-value	*g*	*N*	*z*	*d*	ln(*c*)	*R*	*p*-value	*N*	A_{max}	D_{max}
Normal	*q=0*	Mean	0.315	0.185	0.994	0.000	0.756	35	0.383	-0.005	0.101	0.998	0.000	35	71	4
		Std. Err.	0.019	0.063	0.005	0.000	0.016		0.046	0.003	0.077	0.002	0.000			
		Min	0.270	0.030	0.971	0.000	0.719		0.258	-0.014	-0.059	0.991	0.000			
		Max	0.358	0.340	0.999	0.000	0.794		0.506	0.002	0.329	0.999	0.000			
	q=1	Mean	0.292	0.159	0.812	0.002	0.773	35	0.375	-0.007	0.056	0.894	0.000	35	57	3
		Std. Err.	0.102	0.355	0.137	0.013	0.085		0.286	0.024	0.340	0.067	0.000			
		Min	0.043	-0.386	0.290	0.000	0.626		-0.320	-0.060	-0.712	0.561	0.000			
		Max	0.459	1.013	0.956	0.097	0.970		1.034	0.049	0.839	0.969	0.003			
	q=2	Mean	0.397	0.139	0.799	0.002	0.677	35	0.517	-0.010	-0.011	0.883	0.000	35	54	5
		Std. Err.	0.137	0.480	0.124	0.013	0.124		0.402	0.034	0.453	0.063	0.000			
		Min	0.067	-0.763	0.265	0.000	0.369		-0.438	-0.099	-1.348	0.603	0.000			
		Max	0.706	1.175	0.956	0.129	0.953		1.714	0.067	0.901	0.967	0.001			
	q=3	Mean	0.431	0.158	0.807	0.001	0.646	35	0.591	-0.013	-0.042	0.886	0.000	35	47	5
		Std. Err.	0.145	0.504	0.121	0.010	0.135		0.411	0.035	0.470	0.059	0.000			
		Min	0.086	-0.842	0.295	0.000	0.286		-0.333	-0.110	-1.502	0.636	0.000			
		Max	0.778	1.244	0.957	0.091	0.938		1.924	0.062	0.870	0.977	0.000			
Subnormal	*q=0*	Mean	0.319	0.169	0.993	0.000	0.753	28	0.400	-0.008	0.080	0.998	0.000	28	52	4
		Std. Err.	0.016	0.049	0.004	0.000	0.014		0.041	0.004	0.057	0.001	0.000			
		Min	0.273	0.064	0.980	0.000	0.717		0.237	-0.016	-0.067	0.993	0.000			
		Max	0.359	0.310	0.999	0.000	0.792		0.505	0.007	0.238	0.999	0.000			
	q=1	Mean	0.249	0.267	0.846	0.000	0.810	28	0.401	-0.014	0.100	0.915	0.000	28	28	3
		Std. Err.	0.074	0.227	0.100	0.000	0.061		0.253	0.024	0.247	0.057	0.000			
		Min	0.103	-0.184	0.554	0.000	0.670		-0.049	-0.069	-0.426	0.706	0.000			
		Max	0.412	0.800	0.977	0.003	0.926		0.981	0.036	0.565	0.990	0.000			
	q=2	Mean	0.309	0.339	0.801	0.000	0.758	28	0.503	-0.018	0.127	0.885	0.001	28	27	4
		Std. Err.	0.102	0.303	0.116	0.002	0.088		0.385	0.036	0.366	0.090	0.005			
		Min	0.090	-0.432	0.469	0.000	0.579		-0.263	-0.101	-0.790	0.470	0.000			
		Max	0.507	0.896	0.965	0.014	0.936		1.435	0.055	0.844	0.989	0.050			
	q=3	Mean	0.319	0.386	0.801	0.000	0.749	28	0.554	-0.022	0.129	0.887	0.000	28	25	4
		Std. Err.	0.103	0.315	0.117	0.002	0.090		0.392	0.037	0.379	0.085	0.004			
		Min	0.096	-0.460	0.464	0.000	0.544		-0.247	-0.108	-0.874	0.497	0.000			
		Max	0.542	0.902	0.976	0.015	0.931		1.538	0.053	0.857	0.986	0.033			
Abnormal	*q=0*	Mean	0.309	0.186	0.992	0.000	0.761	33	0.391	-0.007	0.088	0.997	0.000	33	58	4
		Std. Err.	0.017	0.056	0.005	0.000	0.014		0.039	0.003	0.059	0.002	0.000			
		Min	0.262	0.053	0.974	0.000	0.726		0.304	-0.016	-0.053	0.992	0.000			
		Max	0.350	0.349	0.999	0.000	0.801		0.497	0.000	0.234	0.999	0.000			
	q=1	Mean	0.248	0.326	0.914	0.000	0.811	33	0.390	-0.012	0.155	0.955	0.000	33	33	3
		Std. Err.	0.060	0.204	0.081	0.000	0.050		0.145	0.012	0.220	0.041	0.000			
		Min	0.088	-0.161	0.567	0.000	0.690		0.076	-0.042	-0.411	0.708	0.000			

		Max	0.389	0.788	0.991	0.001	0.937		0.827	0.015	0.660	0.993	0.000			
	q=2	Mean	0.426	0.288	0.911	0.000	0.654	33	0.540	-0.010	0.150	0.940	0.000	33	57	6
		Std. Err.	0.102	0.327	0.067	0.000	0.095		0.277	0.022	0.364	0.047	0.000			
		Min	0.201	-0.477	0.549	0.000	0.418		0.001	-0.071	-0.892	0.688	0.000			
		Max	0.662	1.167	0.988	0.001	0.850		1.259	0.043	0.828	0.988	0.000			
	q=3	Mean	0.530	0.195	0.913	0.000	0.551	33	0.563	-0.003	0.156	0.936	0.000	33	210	13
		Std. Err.	0.120	0.365	0.057	0.000	0.120		0.339	0.027	0.422	0.052	0.000			
		Min	0.254	-0.601	0.696	0.000	0.268		-0.099	-0.074	-1.055	0.696	0.000			
		Max	0.792	1.020	0.987	0.000	0.808		1.380	0.053	0.881	0.989	0.000			
Combined	*q=0*	Mean	0.265	0.296	0.991	0.000	0.798	96	0.332	-0.002	0.163	0.998	0.000	96	146	4
		Std. Err.	0.012	0.052	0.004	0.000	0.010		0.025	0.001	0.066	0.001	0.000			
		Min	0.225	0.181	0.977	0.000	0.777		0.267	-0.004	-0.018	0.992	0.000			
		Max	0.290	0.460	0.998	0.000	0.831		0.410	-0.001	0.374	0.999	0.000			
	q=1	Mean	0.154	0.508	0.772	0.012	0.886	96	0.273	-0.004	0.272	0.879	0.000	96	68	3
		Std. Err.	0.066	0.292	0.203	0.069	0.051		0.127	0.004	0.307	0.115	0.001			
		Min	-0.010	-0.187	0.071	0.000	0.776		0.031	-0.015	-0.467	0.329	0.000			
		Max	0.292	1.137	0.981	0.496	1.007		0.642	0.005	0.960	0.988	0.005			
	q=2	Mean	0.217	0.631	0.757	0.007	0.835	96	0.385	-0.006	0.298	0.868	0.001	96	68	5
		Std. Err.	0.095	0.427	0.196	0.048	0.077		0.186	0.006	0.443	0.106	0.006			
		Min	0.015	-0.323	0.079	0.000	0.666		0.006	-0.021	-0.787	0.242	0.000			
		Max	0.416	1.520	0.971	0.449	0.990		0.884	0.008	1.107	0.980	0.063			
	q=3	Mean	0.265	0.622	0.784	0.002	0.796	96	0.434	-0.006	0.286	0.869	0.002	96	76	6
		Std. Err.	0.101	0.452	0.164	0.020	0.084		0.215	0.007	0.493	0.109	0.019			
		Min	0.039	-0.368	0.132	0.000	0.600		-0.050	-0.022	-0.839	0.188	0.000			
		Max	0.485	1.512	0.977	0.203	0.972		0.954	0.009	1.245	0.977	0.190			

Table S3. The results from permutation tests of the Alpha-DAR model parameters between the healthy and diseased treatments.

Pairs of treatments	DAR	*Diversity Order*	Paremeter	Normal (N)	Subnormal (S)	Delta Δ`=|N-S|	Permutated Mean |Δ|	SD of |Δ|	*p*-value
Normal *vs.* Subnormal	PL	*q=0*	*z*	0.338	0.355	0.017	0.059	0.045	0.802
			ln(*c*)	4.980	4.985	0.005	0.189	0.143	0.986
		q=1	*z*	0.149	0.130	0.019	0.112	0.085	0.900
			ln(*c*)	2.982	3.136	0.154	0.382	0.302	0.745
		q=2	*z*	0.139	0.089	0.05	0.157	0.118	0.804
			ln(*c*)	2.058	2.280	0.222	0.529	0.413	0.714
		q=3	*z*	0.119	0.061	0.058	0.162	0.120	0.793
			ln(*c*)	1.746	1.982	0.236	0.538	0.416	0.714
	PLEC	*q=0*	*z*	0.417	0.454	0.037	0.115	0.090	0.803
			d	-0.007	-0.011	0.004	0.007	0.005	0.615
			ln(*c*)	4.902	4.904	0.002	0.235	0.178	0.994
			MAD	479	463	16	108.887	175.968	0.911
		q=1	*z*	0.152	0.219	0.067	0.239	0.174	0.840
			d	0.000	-0.010	0.01	0.013	0.011	0.558
			ln(*c*)	2.979	3.064	0.085	0.475	0.354	0.888
			MAD	45	34	11	6.680	7.509	0.202
		q=2	*z*	0.104	0.190	0.086	0.324	0.246	0.823
			d	0.003	-0.011	0.014	0.021	0.016	0.566
			ln(*c*)	2.092	2.199	0.107	0.586	0.445	0.881
			MAD	NA	13	NA	4.076	3.354	NA
		q=3	*z*	0.084	0.177	0.093	0.330	0.251	0.799
			d	0.003	-0.013	0.016	0.022	0.017	0.577
			ln(*c*)	1.780	1.888	0.108	0.575	0.434	0.878
			MAD	NA	9	NA	3.115	2.316	NA
**Pairs of treatments**	**DAR**	***Diversity Order***	**Paremeter**	**Normal (N)**	**Abnormal (A)**	**Delta Δ`=|N-A|**	**Permutated Mean |Δ|**	**SD of |Δ|**	***p*-value**
Normal *vs.* Abnormal	PL	*q=0*	*z*	0.338	0.332	0.006	0.062	0.046	0.953
			ln(*c*)	4.980	5.047	0.067	0.196	0.145	0.787
		q=1	*z*	0.149	0.150	0.001	0.113	0.088	0.993
			ln(*c*)	2.982	3.152	0.17	0.394	0.314	0.715
		q=2	*z*	0.139	0.122	0.017	0.159	0.120	0.934

			ln(*c*)	2.058	2.335	0.277	0.544	0.424	0.677
		q=3	*z*	0.119	0.101	0.018	0.165	0.123	0.933
			ln(*c*)	1.746	2.031	0.285	0.557	0.426	0.673
	PLEC	*q=0*	*z*	0.417	0.429	0.012	0.117	0.088	0.949
			d	-0.007	-0.009	0.002	0.007	0.006	0.810
			ln(*c*)	4.902	4.957	0.055	0.236	0.173	0.858
			MAD	479	478	1	112.877	139.089	0.998
		q=1	*z*	0.152	0.236	0.084	0.241	0.186	0.776
			d	0.000	-0.008	0.008	0.015	0.012	0.668
			ln(*c*)	2.979	3.072	0.093	0.464	0.357	0.880
			MAD	45	38	7	7.343	8.916	0.403
		q=2	*z*	0.104	0.195	0.091	0.344	0.257	0.832
			d	0.003	-0.007	0.01	0.023	0.018	0.708
			ln(*c*)	2.092	2.267	0.175	0.594	0.456	0.822
			MAD	NA	15	NA	4.384	3.630	NA
		q=3	*z*	0.084	0.171	0.087	0.350	0.261	0.839
			d	0.003	-0.007	0.01	0.024	0.018	0.750
			ln(*c*)	1.780	1.966	0.186	0.579	0.443	0.795
			MAD	NA	10	NA	3.222	2.299	NA
**Pairs of treatments**	**DAR**	***Diversity Order***	**Paremeter**	**Subnormal (S)**	**Abnormal (A)**	**Delta Δ\`=|S-A|**	**Permutated Mean |Δ|**	**SD of |Δ|**	***p*-value**
Subnormal *vs.* Abnormal	PL	*q=0*	*z*	0.355	0.332	0.023	0.192	0.147	0.781
			ln(*c*)	4.985	5.047	0.062	0.061	0.047	0.744
		q=1	*z*	0.130	0.150	0.02	0.391	0.310	0.971
			ln(*c*)	3.136	3.152	0.016	0.113	0.086	0.889
		q=2	*z*	0.089	0.122	0.033	0.526	0.412	0.934
			ln(*c*)	2.280	2.335	0.055	0.152	0.116	0.863
		q=3	*z*	0.061	0.101	0.04	0.534	0.413	0.940
			ln(*c*)	1.982	2.031	0.049	0.157	0.118	0.837
	PLEC	*q=0*	*z*	0.454	0.429	0.025	0.114	0.090	0.848
			d	-0.011	-0.009	0.002	0.007	0.006	0.816
			ln(*c*)	4.904	4.957	0.053	0.231	0.178	0.844
			MAD	463	478	15	110.044	145.802	0.928
		q=1	*z*	0.219	0.236	0.017	0.234	0.172	0.957
			d	-0.010	-0.008	0.002	0.015	0.012	0.931
			ln(*c*)	3.064	3.072	0.008	0.457	0.351	0.988
			MAD	34	38	4	7.029	7.271	0.666
		q=2	*z*	0.190	0.195	0.005	0.327	0.238	0.994
			d	-0.011	-0.007	0.004	0.022	0.017	0.882
			ln(*c*)	2.199	2.267	0.068	0.556	0.433	0.914
			MAD	13	15	2	4.518	4.209	0.665
		q=3	*z*	0.177	0.171	0.006	0.332	0.242	0.983
			d	-0.013	-0.007	0.006	0.023	0.017	0.824
			ln(*c*)	1.888	1.966	0.078	0.545	0.424	0.902
			MAD	9	10	1	3.359	2.579	0.716

Table S4. The results from permutation tests of the B-DAR model parameters between the normal and abnormal treatments

**Pairs of Treatments**	**DAR**	**Diversity Order**	**Parameter**	**Normal (N)**	**Subnormal (S)**	**Delta Δ\`=|N-S|**	**Permutated Mean |Δ|**	**SD of |Δ|**	***p*-value**
Normal *vs.* Subnormal	PL	*q=0*	*z*	0.315	0.319	0.004	0.029	0.021	0.910
			ln(*c*)	0.185	0.169	0.016	0.081	0.064	0.864
		q=1	*z*	0.292	0.249	0.043	0.096	0.074	0.721
			ln(*c*)	0.159	0.267	0.108	0.305	0.229	0.774
		q=2	*z*	0.397	0.309	0.088	0.147	0.114	0.619
			ln(*c*)	0.139	0.339	0.2	0.448	0.332	0.730
		q=3	*z*	0.431	0.319	0.112	0.174	0.132	0.607
			ln(*c*)	0.158	0.386	0.228	0.494	0.364	0.716
	PLEC	*q=0*	*z*	0.383	0.400	0.017	0.048	0.035	0.780
			d	-0.005	-0.008	0.003	0.003	0.003	0.597
			ln(*c*)	0.101	0.080	0.021	0.079	0.058	0.837
			MAD	4	4	0	0.665	0.836	0.701
		q=1	*z*	0.375	0.401	0.026	0.193	0.151	0.918
			d	-0.007	-0.014	0.007	0.014	0.011	0.620
			ln(*c*)	0.056	0.100	0.044	0.300	0.220	0.918
			MAD	3	3	0	0.718	0.719	0.557

		q=2	*z*	0.517	0.503	0.014	0.314	0.246	0.979
			d	-0.010	-0.018	0.008	0.023	0.018	0.760
			ln(*c*)	-0.011	0.127	0.138	0.456	0.326	0.820
			MAD	5	4	1	1.710	2.161	0.588
		q=3	*z*	0.591	0.554	0.037	0.355	0.280	0.923
			d	-0.013	-0.022	0.009	0.026	0.021	0.746
			ln(*c*)	-0.042	0.129	0.171	0.502	0.371	0.802
			MAD	5	4	1	2.546	3.976	0.655
**Pairs of Treatments**	**DAR**	***Diversity Order***	**Parameter**	**Normal (N)**	**Abnormal (A)**	**Delta Δ`=|N-A|**	**Permutated Mean |Δ|**	**SD of |Δ|**	***p*-value**
Normal *vs.* Abnormal	PL	*q=0*	*z*	0.315	0.309	0.006	0.031	0.021	1.000
			ln(*c*)	0.185	0.186	0.001	0.084	0.060	1.000
		q=1	*z*	0.292	0.248	0.044	0.098	0.074	1.000
			ln(*c*)	0.159	0.326	0.167	0.309	0.237	1.000
		q=2	*z*	0.397	0.426	0.029	0.151	0.114	1.000
			ln(*c*)	0.139	0.288	0.149	0.458	0.348	1.000
		q=3	*z*	0.431	0.530	0.099	0.176	0.134	1.000
			ln(*c*)	0.158	0.195	0.037	0.505	0.382	1.000
	PLEC	*q=0*	*z*	0.383	0.391	0.008	0.048	0.035	1.000
			d	-0.005	-0.007	0.002	0.003	0.003	1.000
			ln(*c*)	0.101	0.088	0.013	0.079	0.058	1.000
			MAD	4	4	0	0.665	0.836	1.000
		q=1	*z*	0.375	0.390	0.015	0.193	0.151	1.000
			d	-0.007	-0.012	0.005	0.014	0.011	1.000
			ln(*c*)	0.056	0.155	0.099	0.300	0.220	1.000
			MAD	3	3	0	0.718	0.719	1.000
		q=2	*z*	0.517	0.540	0.023	0.314	0.246	1.000
			d	-0.010	-0.010	0	0.023	0.018	1.000
			ln(*c*)	-0.011	0.150	0.161	0.456	0.326	1.000
			MAD	5	6	1	1.710	2.161	1.000
		q=3	*z*	0.591	0.563	0.028	0.355	0.280	1.000
			d	-0.013	-0.003	0.01	0.026	0.021	1.000
			ln(*c*)	-0.042	0.156	0.198	0.502	0.371	1.000
			MAD	5	13	8	2.546	3.976	1.000
**Pairs of Treatments**	**DAR**	***Diversity Order***	**Parameter**	**Subnormal (S)**	**Abnormal (A)**	**Delta Δ`=|S-A|**	**Permutated Mean |Δ|**	**SD of |Δ|**	***p*-value**
Subnormal *vs.* Abnormal	PL	*q=0*	*z*	0.319	0.309	0.01	0.032	0.021	0.944
			ln(*c*)	0.169	0.186	0.017	0.085	0.060	0.884
		q=1	*z*	0.249	0.248	0.001	0.103	0.077	0.736
			ln(*c*)	0.267	0.326	0.059	0.307	0.233	0.784
		q=2	*z*	0.309	0.426	0.117	0.158	0.117	0.657
			ln(*c*)	0.339	0.288	0.051	0.449	0.342	0.716
		q=3	*z*	0.319	0.530	0.211	0.181	0.135	0.615
			ln(*c*)	0.386	0.195	0.191	0.492	0.370	0.709
	PLEC	*q=0*	*z*	0.400	0.391	0.009	0.049	0.036	0.790
			d	-0.008	-0.007	0.001	0.079	0.062	0.824
			ln(*c*)	0.080	0.088	0.008	0.003	0.003	0.590
			MAD	4	4	0	0.634	0.821	0.687
		q=1	*z*	0.401	0.390	0.011	0.200	0.150	0.918
			d	-0.014	-0.012	0.002	0.014	0.011	0.599
			ln(*c*)	0.100	0.155	0.055	0.322	0.225	0.918
			MAD	3	3	0	0.752	0.715	0.592
		q=2	*z*	0.503	0.540	0.037	0.324	0.238	0.971
			d	-0.018	-0.010	0.008	0.022	0.018	0.716
			ln(*c*)	0.127	0.150	0.023	0.471	0.326	0.815
			MAD	4	6	2	1.743	2.863	0.613
		q=3	*z*	0.554	0.563	0.009	0.369	0.271	0.945
			d	-0.022	-0.003	0.019	0.025	0.020	0.714
			ln(*c*)	0.129	0.156	0.027	0.512	0.369	0.788
			MAD	4	13	9	2.381	2.576	0.666
